# Contraceptive counselling experiences in Spain in the process of creating a web-based contraceptive decision support tool: a qualitative study

**DOI:** 10.1186/s12978-021-01254-0

**Published:** 2021-11-27

**Authors:** Laura Reyes-Martí, Lourdes Rubio-Rico, Laura Ortega-Sanz, Laia Raigal-Aran, Miriam de la Flor-López, Alba Roca-Biosca, Francesc Valls-Fonayet, Montse Moharra-Francés, Ramon Escuriet-Peiro, María Inmaculada de Molina-Fernández

**Affiliations:** 1 Nursing Department, Universitat Rovira i Virgili, Av/Catalunya, 35, 43002 Tarragona, Spain; 2 Medicine Department, Universitat Rovira i Virgili, C/Dr. Mallafrè Guasch, 4, 43005 Tarragona, Spain; 3 Agency for Health Quality and Assessment of Catalonia (AQuAS) of the Catalan Ministry of Health, Carrer de Roc Boronat, 81, 08005 Barcelona, Spain; 4 Catalan Health Service of the Catalan Ministry of Health, Travessera de Les Corts, 131-159 - Edifici Olímpia. Població, 08028 Barcelona, Spain

**Keywords:** Contraceptive counselling, Shared decision support tools, Qualitative research methods, Contraception, Family planning, Adherence, Asesoramiento anticonceptivo, Toma de decisiones compartida, Métodos de investigación cualitativa, Anticoncepción, Planificación familiar, Adherencia

## Abstract

**Background:**

The choice of contraceptive method is a complex decision, and professionals should offer counselling based on the preferences, values and personal situation of the user(s). Some users are unsatisfied with the counselling received, which may, among other consequences, adversely affect method use adherence. In view of this situation, we propose exploring the experiences and needs of users and professionals for contraceptive counselling, in the context of creating a web-based contraceptive decision support tool.

**Methods/design:**

Qualitative research was conducted through focus group discussions (64 users split into eight groups, and 19 professionals in two groups, in Tarragona, Spain) to explore the subjects’ experiences and needs. The data were categorized and the categories were defined and classified based on the three-step protocol or framework for Quality on Contraceptive Counseling (QCC), created by experts, which reviews the quality of interactions between user and professional during the counselling process.

**Results:**

In counselling, users demand more information about the different methods, in an environment of erroneous knowledge and misinformation, which lead to false beliefs and myths in the population that are not contrasted by the professional in counselling. They complain that the method is imposed on them and that their views regarding the decision are not considered. Professionals are concerned that their lack of training leads to counselling directed towards the methods they know best. They acknowledge that a paternalistic paradigm persists in the healthcare they provide, and decision support tools may help to improve the situation.

**Conclusions:**

Users feel unsatisfied and/or demand more information and a warmer, more caring approach. Professionals are reluctant to assume a process of shared decision-making. The use of a contraception DST website may solve some shortcomings in counselling detected in our environment.

## Plain English summary

In Spain, individuals can use a variety of contraceptive methods. Professionals involved in contraceptive counselling should help users to choose and properly use the method that best suits their preferences, personal situation and health status. However, there is a high number of unplanned pregnancies and the population feels dissatisfied with the counselling received. In the context of creating a website dealing with contraceptive methods, we wished to find out how counselling was taking place, giving voice those directly concerned. A qualitative study was proposed to explore the experiences and needs of users and professionals during counselling. Ten focus groups were conducted involving 64 users and 19 professionals from Tarragona (Spain). The results were ordered based on the Quality on Contraceptive Counseling (QCC) framework, created by experts, which determines what the relationship between user and professional should be like during the counselling process. Our analysis highlighted users’ little knowledge and false beliefs concerning contraception who, moreover, complained of receiving little information during counselling. They expressed their discontent with methods being imposed on them by the professional without exploring their preferences or needs. Meanwhile, the professionals argued that they often prescribe the method with which they are most familiar due to the lack of time during consultations at their office and/or because they are unfamiliar with other methods. These results, among others, offer the possibility of improving contraceptive counselling in Spain, if solutions to the difficulties and/or barriers detected are posed and implemented.

## Resumen

**Antecedentes:**

La elección del método anticonceptivo es una decisión compleja y los profesionales deben ofrecer un asesoramiento en base a las preferencias, valores y situación personal de la/os usuaria/os. Hay usuaria/os insatisfecha/os ante el asesoramiento recibido, lo que puede afectar negativamente a la adherencia, entre otros. Ante esta situación se plantea explorar las experiencias y necesidades de usuaria/os y profesionales durante el asesoramiento en anticoncepción, en el contexto de creación de una Herramienta web, de Ayuda a la Toma de Decisiones en anticoncepción.

**Metodología:**

Se llevó a cabo una investigación cualitativa en Tarragona, a partir de 8 grupos focales de 64 usuaria/os y 2 grupos focales de 19 profesionales implicados en el asesoramiento anticonceptivo. Los datos se categorizaron y las categorías se definieron y clasificaron en base al protocolo en tres etapas, Quality on Contraceptive Counseling (QCC), creado por expertos, y que revisa la calidad de las interacciones entre el usuario y el profesional durante el proceso de asesoramiento.

**Resultados:**

Las/os usuarias/os demandan más información en consulta sobre los diferentes métodos, en un entorno de conocimientos erróneos y desinformación, que favorece las falsas creencias y mitos en la población, que no son contrastados por el profesional. Se quejan de la imposición del método, por parte del clínico, en un entorno que no favorece, la toma de decisiones compartidas. Los profesionales se muestran preocupados porque su falta de formación provoque un asesoramiento dirigido hacia los métodos que mejor conocen. Reconocen que persiste un paradigma paternalista en la atención, y que las Herramientas de Ayuda a la Toma de decisiones pueden ayudar a mejorar la situación.

**Conclusiones:**

La/os usuaria/os se sienten insatisfecha/os y demandan más información y una actitud cercana. Hay resistencia por parte de los profesionales a asumir un proceso de toma de decisiones compartida. La utilización de la HATD web “Anticoncepción”, puede resolver parte de los déficits de asesoramiento detectados en nuestro entorno.

## Resumen sencillo en Español

En España, la población puede utilizar una gran variedad de métodos anticonceptivos. Los profesionales, implicados en el asesoramiento anticonceptivo, deben ayudar a que la/os usuarios elijan y utilicen adecuadamente, el método que mejor se adapte a sus preferencias, situación personal y estado de salud. Sin embargo, hay un elevado número de embarazos no deseados y la población se siente insatisfecha ante el asesoramiento recibido. En el contexto de creación de una web sobre métodos anticonceptivos, se quiso conocer cómo se desarrollaba el asesoramiento, dando voz a sus protagonistas. Se planteó una investigación cualitativa, para explorar experiencias y necesidades de usuarios y profesionales, durante el asesoramiento. Se realizaron 10 grupos focales, y participaron 64 usuaria/os y 19 profesionales, de Tarragona (España). Los resultaron se ordenaron en base al Quality on Contraceptive Counseling (QCC), creado por expertos, que determina como debe ser la relación usuari/oa/profesional durante el asesoramiento. Los resultados mostraron los escasos conocimientos y las falsas creencias sobre anticoncepción de las usuarias, que, a su vez, se quejaban de recibir poca información sobre los diferentes métodos en la consulta. Mostraban su desacuerdo a que fuera el profesional quien les impusiera el método, sin explorar sus preferencias o necesidades ante la utilización de este. Por su parte, los profesionales argumentaban que a menudo prescribían el método que mejor conocían, por la falta de tiempo en consulta, y/o por no conocer bien otros métodos. Estos resultados, entre otros, ofrecen la posibilidad de mejorar el asesoramiento anticonceptivo en España, si se plantean e implementan soluciones a las dificultades y/o barreras detectadas.

## Background

In Spain some 190,000 unplanned pregnancies are recorded each year, accounting for 35% of the total, as a result of not use of a contraceptive method or a failure to use it [1]. The current trend in our country is that approximately half of these pregnancies are voluntarily interrupted. A total of 99,149 were reported in 2019 with the physical, emotional and economic consequences this entails [2].

According to the National Survey on contraception in Spain, conducted in 2020 among 1800 women between 15 and 49 years of age, condoms are the method most frequently. Used by 31.3% of couples, followed in frequency by combined oral contraception (COC); by 18.5% of users. These figures are high, considering that 29.3% of women who have sexual relations report not using any method [3].

The condom is a good method of protection against sexually transmitted infections (STIs), but it is ineffective as a contraceptive. Compared to other methods, it is inconsistent and/or it is incorrectly used [3]. According to the Pearl Index, during the first year of use, 15 out of every 100 women who use condoms become pregnant, compared to eight out of every 100 women who use COC [4].

Given the data presented, the Spanish Society of Contraception (SEC) recommends the use of highly effective methods, Long-Acting Reversible Contraception (LARC), a set of methods that includes intrauterine devices (IUD) and the subdermal implant. These long-acting methods have a Pearl Index of less than two pregnancies per 100 users/year, as their contraceptive efficacy is not dependent on their correct use by the user, and are now used by 9.7% of users [3, 4].

Therefore, in recent years, directed contraceptive counselling models have been promoted. As well direct promotion of highly effective specific methods, or a model of “staggered effectiveness”, structuring counselling according to the effectiveness of the methods [5, 6]. However, in a recent publication Dehlendorf has questioned and criticized the suitability of these models of counselling based solely on the concept of effectiveness. It says that it does not take into account the different perspectives that women have about an unwanted pregnancy, or their preferences regarding methods [7]. In this regard, some authors point out that family planning services promote access to and the adoption of safe contraceptive methods. Those are preferably measured numerically, paying little attention to the experiences of the user(s) during counselling [8]. Considering that efficacy is not the only feature, and neither is it always the most important when choosing a contraceptive method [9–11].

The choice of method is a complex decision. It is recommended that professionals offer users focused on making informed decisions about their fertility and on the use of methods in accordance with their reproductive preferences and objectives [7]. The decision as to which method to use is influenced by the values and preferences of the individual, cost impact, subject knowledge, and the advice received, among others [12]. Counselling is said to be one of the most important interventions to improve contraceptive use and adherence [13].

A recent study founds that some women are dissatisfied with the contraceptive counselling they receive. It highlights a scenario in which, predominantly, method choice is heavily influenced by clinician recommendation or imposition [13]. This situation can negatively affect adherence to the method and the quality care provided, as it prevents individuals from making the best decision for themselves [8].

In 2017 Holt et al. presented a three-step protocol or framework for quality on contraceptive counseling (QCC). The aim of this framework, based on experts’ opinions is the application of concepts from the literature on communication in healthcare (respect, empathy, trust). Moreover by identifying the basic principles of human rights with regard to interactions between user and clinician (confidentiality, trust, non-discrimination) [8]. This QCC framework was included in the project to create the recently published the Quality of Contraceptive Counseling Scale [14].

Along these lines, and in order to improve communication during counselling, the development of decision support tools has been encouraged: a set of complementary interventions used, among others, to support clinicians when informing and/or counselling patients [15]. However, there is no evidence that their use improves the quality of healthcare or shared decision-making [16]. One possible reason is the lack of research on the use of these decision support tools and on what women perceive in their interactions with the practitioners during the process [17].

The purpose of this research is to explore the experiences and needs of users and practitioners during contraceptive counselling taking the Quality of Contraceptive Counselling Framework. Holt [8] will be used as a reference, in the context of creating a decision support tool for contraception.

## Materials and methods

### Rationale and context of the research

The study presented is part of the process to design and develop “*Anticoncepción*” (contraception) [18], a decision support tool that has been developed following the stages described by the Network of Agencies for Assessing National Health System Technologies and Performance of the Spanish National Health System [15], recently published in Spanish and Catalan on the “*Decisiones compartidas*” (shared decisions) website of the Agency for Health Quality and Assessment of Catalonia (AQuAS) of the Catalan Ministry of Health [19].

### Study design

In order to explore the experiences of users and practitioners during contraceptive counselling, during the first phase of the contraception decision support tool (DST) development process*,* qualitative research was conducted through focus groups (FG), and the contents of the subsequent transcripts were analysed.

### Subject selection and recruitment

Focus group participants (both users and practitioners) were recruited through the professional network of some of the researchers. Convenience sampling was performed based on different recruitment strategies, such as snowball, WhatsApp groups and email lists. The characteristics of recruitment determined that it was only possible to include respondents who agreed to participate in the study. Those who did not respond were deemed to have refused to participate. All participants with whom an appointment was agreed, attended it.

All participants resided in the province of Tarragona, in southern Catalonia (Spain), in rural and urban environments. Different inclusion criteria were defined for each FG (see Table 1).

**Table 1:** Specific inclusion criteria for the user FG based on age and sex

Young women FG (YW)	Adult women FG (AW)	Men FG (M)	Adolescents (A)
Aged 18 to 30 Sex: female	Aged 30 years and over Sex: female	Aged 18 years and over Sex: male	Aged from 16 to 20 years Sex: female and male
Health service users	Health service users		
Experience or perceived need for contraceptive counselling	Experience or perceived need for contraceptive counselling	Experience or perceived need for contraceptive counselling	Experience or perceived need for contraceptive counselling

For the make-up of user FG, intragroup homogeneity and intergroup heterogeneity were sought in order to achieve a greater diversity of discourse, while avoiding bringing together participants that might hinder group interaction, due to reasons of age and/or sexes, as “sexuality” remains “taboo” for many individuals.

The FG of practitioners included nurses, family physicians, midwives, gynaecologists and social educators working in primary care, hospitals or social services in Tarragona, who provided contraceptive counselling in their work environment.

Prior to participating, users and practitioners read the information sheet containing information about the study objectives and design, and information concerning the names and professional affiliations of the researchers. They then proceeded to sign give informed consent by signing the relevant form.

### Planning and conducting the FG

It was initially planned to hold six FG with women of different ages and two with various practitioners, with between six and 12 people, but the first level of analysis seeking emerging topics highlighted the need to expand the sample to a further two FG of users, one of adolescents of both sexes, and one of men. Following the criteria of Mayan on data saturation, collection continued until the time when it was considered that the data available afforded a new and plausible explanation for the phenomenon studied [20].

The FG were held between 2017 and 2020 in the Board Room of the Department of Nursing, except for the FG with adolescents (July 2020), held in a park in Tarragona, outdoors, due to COVID-19 restrictions.

The analysis of the results of the first FG, allowed to elaborate, a pilot DST in hormonal contraception, at the request of the Agency for Health Quality and Assessment of Catalonia (AQuAS) of the Catalan Ministry of Health, which was tested and published on its website “Decisiones compartidas” in August 2018. This previous analysis, allowed the discovery of information gaps and, later on, new recruitment and data collection actions. The final analysis of the results of all the FG allowed the publication of the DST “Anticoncepción” including hormonal methods, non-hormonal methods, on the website “Decisiones compartidas” of the Departament de Salut de la Generalitat de Catalunya (March 2021) [19].

The FG were coordinated by a **moderator** and an **assistant** of the team of Ph.D-qualified female professors at Rovira i Virgili University (URV), with experience in leading FG who were not known by the participants. Moderators and assistants conducted reflexivity exercises to consider their positionality and the power dynamics for use in the FG. **The assistant** handed out the “ad-hoc” questionnaires for filling in the data on sociodemographic variables and clinical variables related to contraception. Subsequently, she observed and took notes that would later be used to assist with transcription and analysis. To stimulate conversation, **the moderator** used open-ended questions with which she encouraged participants to talk and interact with each other (see Tables 2 and 3).

**Table 2:** Script for the questions for the user focus groups

Think about the times you have requested contraceptive counselling and tell us… what was it like? how did you feel?, what did you feel was missing and why?
Tell us how you made your decision regarding which contraceptive method to use? What influenced you and why?
We would like to know which resources you need and/or use to get information and your experience in this respect, in the consultancy of the practitioner or outside it
Think about the aspects you would like to find out about the different methods of contraception and tell us them
Do doubts and/or fears arise concerning the use of some methods? Which ones? Concerning what?
Do you think a reliable web space containing information that you can access freely from home would help you choose the method? What would you like it to be like?

**Table 3:** Script for the questions for the practitioner focus groups

Please explain how you explore the knowledge, beliefs and/or doubts users have about the various methods
Tell us how you usually resolve a request for counselling
Think about the times you have provided contraceptive counselling and tell us… what was it like?, how did you feel during the visit?
In your view, what aspects do you think users take into account when considering that they have been given good counselling?
Please explain how you think the practitioner should or should not influence the choice of method
What do you think a web-format decision support tool for contraception could bring to practitioners and users?

During the FG, participants’ privacy and confidentiality were ensured, and only the moderator, assistant and participants were present.

The sessions were recorded digitally (Olympus VN-3500PC) and transcribed literally by the researchers in Spanish. The FG lasted between 45 and 60 min.

### Data analysis

A thematic analysis of the contents was carried out. Each FG was analysed, first individually and then jointly, by the researchers, using “*a method to identify, analyse and extract patterns (themes) within the group*”, following the stages of Braun and Clarke [21].

**Fig. 1:**
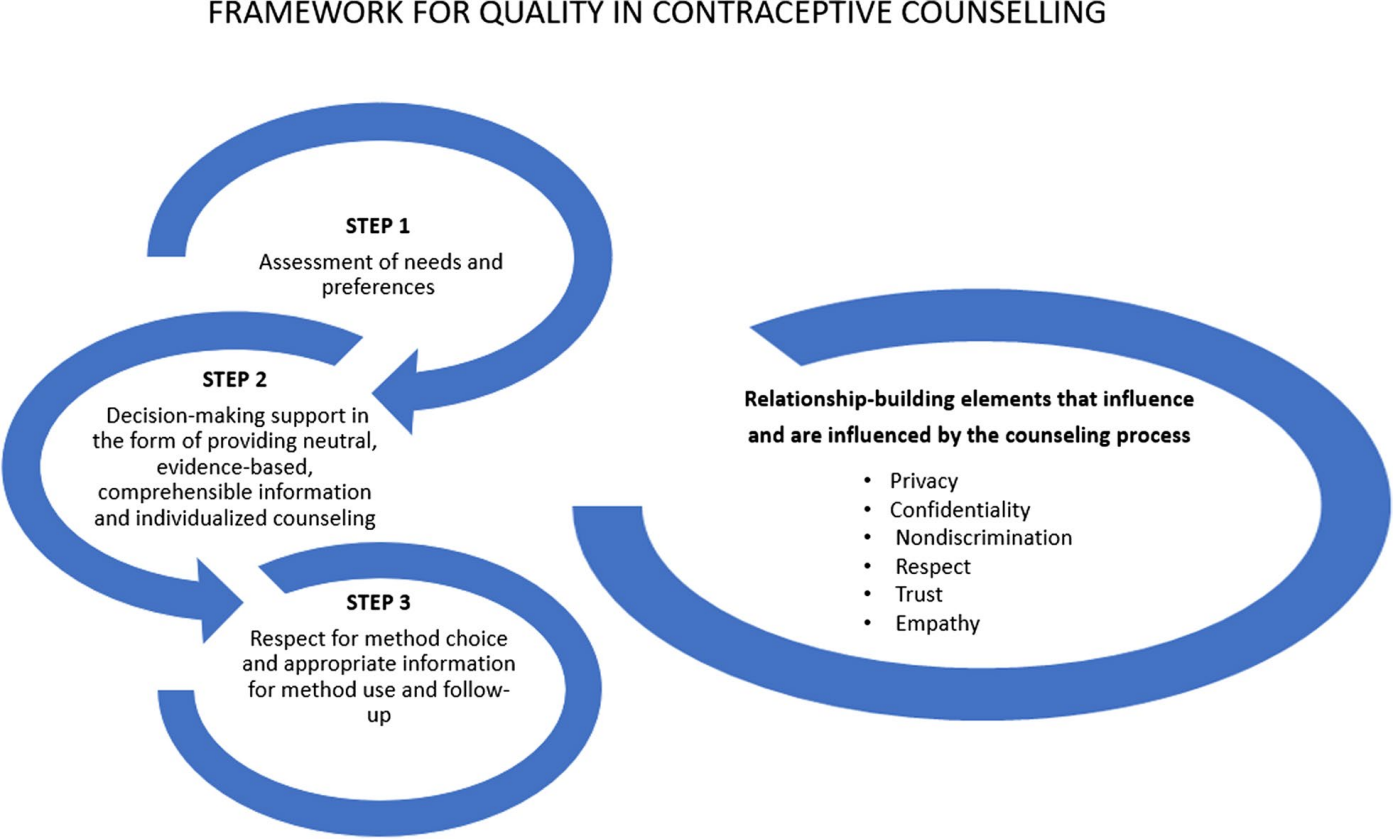
Authors’ own based on “Quality in contraceptive counseling”, taken from the article “Defining quality in contraceptive counseling to improve measurement of individuals’ experiences and enable service delivery improvement” [8]

The data were analysed by five of the researchers. The reporting units were identified and coded using open coding. All transcripts were coded by ARB and MMF using the software package weft-QDA. Each code was reviewed by another member of the team and discrepancies were discussed and resolved. Then, they were categorized by LRA, LRM and REP through a flexible process of expanding and deleting codes based on their content. The categories were then defined and classified into one of the steps of the comprehensive counselling framework (QCC) defined by Holt [8] (see Fig. 1). All analysis was done in Spanish and the direct quotes are translated to English in the “Results” section.

To ensure their validity, after the data analysis was completed, the final analytical topics were presented to the participants for content verification or suggestions. Participants who contacted the researchers did it to say that they were agreed with the analysis themes presented.

### Ethical considerations

The Ethics Committee of the Pere Virgili Health Research Institute (IISPV), Tarragona (Spain), gave its approval to conduct the study “HERRAMIENTA DE AYUDA EN LA TOMA DE DECISIONES EN ANTICONCEPCION” in two phases (1st Phase: Ref. CEIm: 034/2017, and 2nd Phase Ref. CEIm: 177/2019).

## Results

The research involved a total of 64 users and 19 clinical practitioners, distributed into three FG of young women (YW), three FG of adult women (AW), one FG of men (M), one FG of adolescents, and two FG of clinical practitioners. The FG were held between March 2017 and July 2020.

Participating users’ sociodemographic and clinical profiles are set out below (see Table 4).

**Table 4:** Profile of participating users according to sociodemographic and clinical variables

	Categories	n (%)
Sex	Female	49 (77%)
	Male	15 (23%)
Age (years)	15–20	19 (30%)
	21–26	13 (20%)
	27–35	8 (13%)
	36–45	13 (20%)
	≥ 46	11 (17%)
Education level	Primary school	6 (9%)
	Compulsory secondary education	5 (8%)
	Lower vocational training	7 (11%)
	Higher vocational training	8 (13%)
	Baccalaureate (upper secondary)	4 (6%)
	University studies	19 (30%)
	Master’s degree, Postgraduate studies, Doctoral degree	13 (20%)
	None of the above	2 (3%)
Stable couple	Yes	11 (17%)
	No	41 (64%)
	NA/DK	12 (19%)
Current contraceptive use	No	18 (28%)
	Yes	46 (72%)
	*Type of contraceptive method*	
	Condom	27 (59%)
	Combined oral contraceptives	10 (22%)
	Vasectomy/tubal ligation	1 (2%)
	Contraceptive ring	1 (2%)
	Natural methods	1 (2%)
	IUD	4 (9%)
	Progestin-only pills	1 (2%)
	Subdermal implant	1 (2%)
Purpose of the contraceptive method	Contraceptive	25 (39%)
	Prevent STI	12 (19%)
	Both	14 (22%)
	Others^a^	2 (3%)
	NA/DK	11(17%)
Incidences or problems with the current contraceptive method	No	27 (42%)
	Yes	26 (41%)
	NA/DK	11 (17%)
Which one? (more than one answer possible)	Breakage	8
	Slip-off	2
	Omission	7
	Inconvenience	3
	Side effects^b^	15
	Cost	2

^a^Other therapeutic purposes: acne, heavy periods or dysmenorrhea

^b^The onset of spider veins, amenorrhea, lower limb pain, headache, and others not specified

The sociodemographic profiles of the practitioners are presented in Table 5.

**Table 5:** Participating practitioners’ profiles

Profile of family planning providers according to sociodemographic and clinical variables	Categories	n (%)
Sex	Female	17 (89%)
	Male	2 (11%)
Age (years)	20–30	4 (21%)
	31–40	2 (10.5%)
	41–50	3 (16%)
	> 50	10 (53%)
Professional category	Family Physicians	5 (26%)
	Gynaecologist	3 (16%)
	Gynaecology resident	1 (5%)
	Midwife	6 (32%)
	Nurse	3 (16%)
	Social Educator	1 (5%)
Currently working at the time of the investigation	Yes	18 (95%)
	No	1 (5%)

### Qualitative results

Table 6 sets out the main categories found, classified on the basis of the steps described by Holt [8]. Then, separately, users’ and practitioners’ most representative emerging issues are presented, providing the quotations that illustrate the main findings.

**Table 6:** Themes/categories (users and practitioners) classified according to the steps of the “Comprehensive Framework for Contraceptive Counseling by Holt [8]

Step 1	Step 2	Step 3
***Explore the needs, preferences and previous experiences with contraceptives, to adapt communication to specific needs and concerns***	***Facilitate decision-making for the choice of method providing up-to-date information on all options: effectiveness, mechanism of action, side effects, contraindications, barriers to use, *etc*., neutrally and comprehensibly***	***Respect the choice of method in a context of shared decision-making, incorporating information for the use of the method and its monitoring***
*User FG categories*		
Various sources of information, sometimes unreliable or not verified	Lack of information about the different methods in relation to their effectiveness, composition, mechanism of action, protection against sexually transmitted infections, cost, etc.)	Practitioner’s reluctance to cede power of decision
Prescribing the method requested by the user, without exploring their needs		User’s reluctance to take the decision
Concern for the side effects of the methods	Distrust in the practitioner upon receiving biased information or a lack of details regarding the methods	Opinions on accessibility to information outside the physician’s office ()
Unexplored misconceptions and myths		
*Practitioners FG categories*		
Choice based on environmental influences	The importance of providing up-to-date information on effectiveness, cost, safety, prevention STI, SE… of the various methods	Predominant paternalistic approach
		Health system that hinders good monitoring
Difficulties in communication with the user (diverse languages and cultures, excessive caseload and lack of knowledge of contraception)	Practitioners’ lack of specific training in contraception, a hindrance to adequate, neutral counselling	Need to improve accessibility to information for practitioners and users (DST)

### Step 1

#### Users

In general, the choice of the method was based on erroneous knowledge or misinformation regarding the methods available in the market, their composition, mechanism of action and effectiveness, among others. Such aspects should have been routinely investigated and/or verified by the clinician in counselling. The cost appeared as a conditioning factor in choosing the method due to users’ low-income or not having their own resources, as would be the case of the youngest subjects.

**Table:** 

***Category: Various sources of information, sometimes unreliable or not verified*** *“… you start to hear something when you go to ESO [years 8–11 of compulsory schooling], a talk. I, for example, confide a lot in my aunt and I’ve heard all the information from her”, M1* *“where does information on contraception circulate? The friend or brother who explained it to me”, M9* *“I, honestly, where I learned most about contraceptives is in the series “The Big House” (…) I’ve also searched on internet…”, A10* *“I find things about contraception on TV or on YouTube, if you look a little, you only see ads and videos about condoms, at least I’ve only seen those. I haven’t seen an advert for the patch or the IUD”, A6* *“I’ve got friends and a cousin, who have already used the rod (subdermal implant), they had said good things about it and… I opted for this method”, YW16*	

Young women (YW) and adult women (AW) usually attended the clinician’s office having already chosen a contraceptive method, generally based on information obtained from their immediate environment, usually from family and friends. The youngest subjects (A), referred to finding information on social networks, TV and on the internet. Teenagers and men were the most misinformed about contraceptive methods*.*

**Table:** 

***Category: Prescribing the method requested by the user, without exploring their needs*** *“I think you can go to the visit with a fixed idea, but if they offer the full range of opportunities out there, you might change… I don’t know, if you know other methods and they are more advantageous for you it’s much better that they tell you everything”, YW9* *“I think you should be given information about all possible methods, not just talk about condoms, but as we said before, about the dual method…”, YW17* *“hormonal contraceptives have a super strong hormonal shot, right?”, A1* *“it’s important to know what it costs, because a lot of people [who] start having sexual relations don’t have their parents’ consent and have no money”, YW4*	

Users expressed their interest in and/or concern with knowing whether the use of certain hormonal methods would affect menstruation and/or cause side effects such as weight gain, headache, mood swings, diminished sex drive, increased hair, the appearance of blemishes on the skin, amenorrhea and intermenstrual bleeding. Such aspects were often not mentioned by the clinician in counselling and are directly related to the reasons reported when requesting a change of method.

**Table:** 

***Category: Concern for the side effects of the methods*** *“We have used birth control pills, but… well… we’ve been reading that they cause hormonal changes, like putting on weight or that they can eventually leave you sterile… And that’s why we decided to change to the condom”, M8* *“…I know lots of people who say that hormones disagree with them. ….I don’t know, since your body doesn’t produce them, they can cause some kind of rejection, I think”, YW17* *“When I used the IUD the first month I didn’t have a period and I was afraid I might be pregnant, now in the second month I’ve had my period, but it bothers me not knowing when I will get it and when it will finish…”, YW18* *“I need to know the side effects… I don’t want to turn into a cold woman… to age quicker, ‘cos that’s got to do with hormones… you know?”, AW22* *“I decided to try a hormonal method, the pill in my case… I noticed it changed my character and that I was in a bad mood… and also blemishes on the skin with the ring… I’ve got blemishes on the skin…”, AW1* *“…with the ring, really great, but I stopped because when having sex my drive decreased, I didn’t feel so much like having relations…”, YW9* *“well they’re hormones and your body changes inside… You can put on weight or you can also lose weight, or the question of hair too… you can have more or less…”, A8*	

Misconceptions and myths were very widespread among the population, and were associated with taking contraceptives and future infertility. For example, the use of the IUD exclusive to women who had been mothers, amenorrhea as something negative for the woman’s body and the need for breaks in taking contraceptives. These aspects were not always explored by the clinician, leading them to persist over time.

**Table:** 

***Category: Unexplored misconceptions and myths*** *“…I’ve been told that if you don’t take a break you become infertile (talking about COC)”, YW5* *“they say that after a few months of taking the pill you should rest for a month or two, then start again…”, YW11* *“Imagine, because if you don’t get your period for five years… you imagine the headaches… you imagine the bad mood… that can’t be normal, it can’t be good… because the lack of oestrogen gives you wrinkles, you suddenly become an old woman, it’s as if you were in menopause (….). and I think, if this is something we need as a woman, I mean, we need to get rid of that blood, as they say “it’s bad”, it’s useless, what happens to all that blood that accumulates there…?”, AW22* *“…that each year of birth control pills are 7 years of ageing of the womb…”, YW4* *“I’ve been told that there was an IUD that wasn’t hormonal but for people who hadn’t had children we should never use it, as there was risk of damaging the uterus”, AW1* *“We have used birth control pills, but we’ve been reading that they cause hormonal changes, like putting on weight, that it can eventually leave you sterile… for women, right? That’s what I understand and that’s why we decided to change to condoms”, M8*	

#### Practitioners

Practitioners reported that women come to their office with the idea of a method based on their age or if they have a partner. They express that even if they agree that they should be advised on other methods and inquire into the subject’s personal and financial situation, their stage of life or experiences with other methods, among others, they do not get involved in the choice. Most agreed that the population lacks knowledge about contraception and that counselling should be individualized, although they rather understand it as informing about a method than how to encourage the choice based on a shared decision.

**Table:** 

***Category: Choice based on environmental influences*** *“take pills, and they do the job. And you try to explain that, well, the IUD might suit her better. People come with an idea already for a contraceptive method. They want pills because their neighbour on the fourth floor takes them and they suit her (…) then it’s very difficult to convince them…”, (Nurse, > 50 years of age)* *“…you have to limit the information according to the characteristics of the patient, her medical condition, and what you think will best suit her… because sometimes what is best for your patient in your medical opinion, is not what she wants…”, (family doctor, > 50 years of age)* *“…that’s why there are so many contraceptive methods, because there are many profiles of women. The first thing I explain is what she asks me for. I make sure she asks me for it because she knows about it, I clear up any doubts and if she is still interested, okay. What I think is that, if she leaves the office with a different method from the one she has decided for, she won’t use it… “, (family doctor, 41–50 years of age)* *“they base themselves on their surroundings, right? their friend, their sister, (…) so there’s a great predisposition towards this or that method, or they want to use one because it works well for such-and-such, and so they are completely unaware of the side effects, or if it’s suitable for her or not “, (Gynaecologist, 31–40 years of age)*	

Practitioners perceived that the users they treat have little knowledge about contraception and difficulties in interpreting a patient information leaflet. This is compounded among immigrant women. Some of the issues they considered would hamper and/or exacerbate this situation would be excessive caseloads, a different language and lack of specific training in contraception for some practitioners.

**Table:** 

***Category: Difficulties in communicating with users (diverse languages and cultures, excessive caseload and lack of knowledge of contraception)*** *“All this is in the patient information leaflet… but for some reason people read patient information leaflets and don’t really understand them… because after an oversight it clearly states there to use a barrier method… but they don’t”, (Family doctor, > 50 years of age)* *“Because now in surgery we have a language barrier… however much you want to give information, there are people who won’t receive it all…”, (Gynaecologist, 20–30 years of age)* *“…you have excessive caseloads and, also, you don’t have specific training… So a visit will take a long time because you are misinformed, therefore, do not know how to inform… the woman has doubts and probably leaves dissatisfied… another problem is the lack of knowledge because there are still practitioners who decide to suspend the contraceptive method to take breaks…”, (Midwife, 20–30 years old)* *“…I always speak from experience from where I work… if you receive a Moroccan or a Chinese woman and she doesn’t understand me… how do I do it? How can I explain how a natural method works?”, (Midwife, > 50 years of age)* *“…I deal a lot with young Moroccans and the information they have about contraception is zero, they know nothing whatsoever, the condom at most…”, (Social educator, 31–40 years of age)*	

### Step 2

#### Users

Users commented that they receive little information on contraception from the practitioners treating them and request more information about the contraceptive methods available in the market, mechanisms of action, protection against STI, their effectiveness or cost, unaware of the possibility of using a subsidized method, among others.

**Table:** 

***Category: Lack of information about the different methods in relation to their effectiveness, composition, mechanism of action, protection against sexually transmitted infections, cost, etc*** *“What I don’t know is if all contraceptives can be used to prevent pregnancy or can also be used to protect against STD. That, I don’t know, don’t know, which have an effect on STD or not… the male condom does, and the female one?”, A6* *“…when I was informed about the available contraceptive methods, I looked above all that it was effective because… although getting pregnant wouldn’t be a drama, I don’t want it to happen…”, YW3* *“if the practitioners tell me that the contraceptive method has a certain percentage of safety, then I’ll think about it… but I want to know…”, AW22* *“I take COC, because it was the only option they gave me, for example I don’t know what the diaphragm is. I think there’s a lack of information. I, for example, nowadays don’t know many of contraceptives there are, I feel uninformed”, AW5* *“…I like the IUD, the thing is that two hundred euros… where do you get it from?”, AW22*	

Sometimes, somewhat neutral advice, accompanied by insufficient information, or the use of overly technical language, leaded to distrust in the practitioner and, ultimately, a bad counselling experience.

**Table:** 

***Category: Distrust in the practitioner upon receiving biased information or a lack of details regarding the methods*** *“I’ve always thought, in view of my dealings with different gynaecologists I’ve visited, that there’s a league in favour of the COC. When I asked about the diaphragm, they told me, ugh, no,, it’s very little effective!” (…), even asking for a method without hormones, they offered me the ring… as if it didn’t have any!”, AW9* *“…he insisted I should use the IUD, and I don’t know why… and I asked him: ‘Is it something commercial?’ what new thing has come out, are you testing it or why are you insisting I should use it?”, AW18* *“At 16 I went to ask about some other contraceptive method (I was already taking birth control pills) and [he/she] said,” Take the pills you are already taking and that’s that” and I left the office without any more information”, YW10* *“…I told the gynaecologist I was studying nursing and then he relaxed a little and explained a little more… because at the beginning it was like he didn’t want to go into the subject very much because he didn’t know how to explain it to me without it being very technical… then… it creates a lack of information in the end”, YW8* *“I think maybe there should be a more thorough examination to find out your conditions, they do it at some (private) centres (…) they give you a blood test and look at your hormone levels, and from there they give you one thing or another”, YW3*	

#### Practitioners

Practitioners believed it is important to properly advise the population. Issues such as cost, safety, effectiveness, the prevention of STI, side effects and others have to be considered when informing as to the different methods. They add that mostly, women decide to change or even cease contraceptive method due to price and SE. Regarding SE, they believe more time should be spent explaining them better, to thus try to reduce abandonment.

**Table:** 

***Category: The importance of providing up-to-date information on effectiveness, cost, safety, prevention STI, SE… of the various methods*** *“…it is essential to explain how it works, how it is used, if it has any side effect… if there is any change… for example, if we talk about the implant, the first thing that is explained is the percentage likelihood of amenorrhea, the percentage likelihood of irregular bleeding…”, (Midwife, > 50 years of age)* *“…I think the key is to spend time explaining things… for example, in the case of the hormonal IUD: that a third of women experience amenorrhea or maybe they get it (menstruation) once or twice a year or have very few periods… and there will be others who, with the copper IUD have prolonged, heavy periods… in short, if you explain it well… women adapt and are more receptive to the method…”, (Midwife, > 50 years of age)* *“Among young people it’s important to explain very well that the condom is not only to prevent pregnancy… the fear that there was of HIV a few years ago has been somewhat forgotten, and I think that that’s also very important in the end”, (Resident gynaecologist, 20–30 years of age)* *“…there have been many users who have changed their method for financial reasons. For example, all users of the vaginal ring, of which there were many and they were very happy, have changed method and have gone to a subsidized oral contraceptive because they can’t afford the cost of the patch or the ring…”, (Gynaecologist, 20–30 years of age)*	

Practitioners were concerned about the fact that their lack of knowledge of some of the methods or lack of specific training, lead to inadequate advice directed towards the methods with which they are most familiar. They considered that more training on the subject would allow them to better advise, in the limited time they have in the office. Caseloads were an argument to excuse their failure to inform of all available methods.

**Table:** 

***Category: Practitioners’ lack of specific training in contraception*** *“…there are practitioners who, faced with a request for combined hormonal contraceptives, because they don’t know, continue to give “Loeb”, “Sibilla” or “Diane 35″, although the guidelines say they shouldn’t be prescribed… this happens because we don’t refresh… and if we ourselves don’t know the characteristics of the methods, how are we going to explain or prescribe them?”, (Midwife, 20–30 years of age)* *“I think that not only are users unaware of contraceptive methods, but among practitioners there is also a lack of information and we end up deciding for them”, (Midwife, > 50 years of age)* *“…Excessive caseloads makes consultations, perhaps, not long enough (…), but, also, you don’t have specific training in this and this will take a long time at the consultation because you are uninformed, you don’t know how to inform, the woman has doubts, you will generate more doubts, it’s never-ending, right?, and probably the woman leaves unsatisfied and you too…”, (Family doctor, > 50 years of age)* *“the user should be given more information when you’re recommending a method or you’re advising her, also explain to her a little beyond how to use that method… there’s a lack of pedagogy…”, (Gynaecologist, 20–30 years of age)*	

### Step 3

#### Users

Some users felt that the practitioner imposes the contraceptive method on them without offering others or that they are not supported when they express their wish to use a particular method, different from the one recommended.

**Table:** 

***Category: Practitioner’s reluctance to cede power of decision*** *“The thing is that the subject of contraceptives is very personal due to opinions, personal experiences and so on… like it gives you a little more independence when you decide (…) not just that the doctor controls what you have to do and what not… There will be doctors who are well intentioned, but in the end it’s still a treatment for you, that, if it suits you or not… they are not affected, so if you don’t get on well with the practitioner, or whatever, then he will give you whatever’s best for him, full stop”, YW8* *“the gynaecologist stopped me on the subject of the IUD… and told me to continue with the pills if I wanted to go on with some method of contraception… she didn’t give me more information about other methods…”, YW13* *“I take these contraceptive pills because they’re the ones my gynaecologist gave me… he told me to take them, but without giving me more options…”, YW9* *“but the issue of ligation… is I when I went to the office I had the feeling that the midwife didn’t support me in my decision, when for me it hasn’t been a decision like “I get up and, ah! I’ll have a ligation!”… for me it has been a super-thought-through decision”, AW19* *“When I got pregnant she gave me what for, called me everything under the sun… I mean, everything but good-looking… until I got angry and said, look, I’m forty-one, my husband got me pregnant and it was an accident…”, AW22*	

However, there were women who felt comfortable and confident that the practitioner can decide for them which method to use.

**Table:** 

***Category: User reluctance to take the decision/Practitioner’s reluctance to cede power of decision*** *“Well, I have a lot of confidence in her and I say: whatever you recommend to me, I’ll take it”, YW9* *“but because I trust her and I know that if she had said it’s no good (contraceptive method), I don’t use it, simple as that…”, YW9* *“in my case, I always ask the gynaecologist… because in my country in Venezuela there’s a great tendency that if you need something you consult your doctor. (…) and until the gynaecologist approves, you don’t buy anything”, YW5*	

In general, they asked to have greater access to the system and/or access to reliable information, outside the physician’s office, to encourage their involvement in the decision-making process and be able to clear up their doubts if they arise during use of the method. Women (AW and YW), consider that good follow-up by practitioners is important and that it could be supported by leaflets, brochures or a reliable, up-to-date website. They displayed a general interest in creating a decision support tool for contraception. It would be important to make suggestions on how they would like it to be, with audiovisual content and little text, first-hand accounts, a virtual assistant and/or freephone for queries. Younger subjects (A) also like the idea of being able to access information via the web, but stress the need for it to be accessible from a smartphone and for access to be available from social networks like Facebook or Instagram.

**Table:** 

***Category: Opinions on accessibility to information outside the physician’s office for the DST*** *“I think there is information, but you have to go to look for it… You don’t have it at your fingertips… you have to get on with it, on your own initiative…”, AW11* *“…(DST) I think the recommendation of a website with first-hand accounts would be interesting (…) also with specific information on the different methods, and videos… that should be short… most essential… less than a minute, I think it’s preferable to separate, ‘pills’ of the basics, it must be easy to see from any mobile device, easy to navigate… a little that, for each method, you had effectiveness, protection, and you decide to be able to compare them… “, AW5* *“…(DST) with drawings that are super clear”, YW5* *“…(DST) with basic ideas and then access to more information”, YW7* *“…(DST) if there is a lot of text it won’t be read…. I’m an odd case, I like reading a lot, (…) people see 4–5 things and run away”, YW2* *“(DST) Talks, not too many. Words, not too many. Picture, film, movie… Two minutes. Three minutes. No more. When you go to the school and you have the little boy after 5 min you’re not talking to him he’s already playing with the one next to him and he doesn’t understand. It must be film. Audiovisual.”, M9* *“(DST) could also be interesting, apart from the thing of the phone, that on that webpage, it offered the possibility of being able to submit a query in writing to a virtual assistant, like some companies do, like IKEA”, AW5* *“…(DST) More than a website, it would be interesting to put information on Instagram with a link to go to the website (…) that’s what’s in nowadays”, A4*	

#### Practitioners

Some practitioners recognized that a paternalistic paradigm persists in healthcare and that often they decide the contraceptive for the woman. The perception of women’s trust in them, as practitioners, was one of the reasons they upheld. In other cases, they justified a higher degree of paternalism in cases where they perceived irresponsibility on the part of the user with respect to their health.

**Table:** 

***Category: Predominant paternalistic approach*** *“…there we are deciding for her, I think, it’s the model we have come from and is still there, it’s hard for us to change, because the tendency we have is in that direction, to decide for her, and you think, how am I going to get you into this mess now?…maybe a “tool of this kind” (DST), is the way to ensure the information gets across”, (Family doctor, > 50 years of age)* *“in any case I think that, deep down, they trust quite a lot in us, the practitioners. Any method that you tell them… because they always tell you, recommend the best one, (…) most are quite receptive to advice. They trust you quite a lot”, (Midwife, > 50 years of age)* *“I think it depends on the woman, on the moment, because, for example, if I have a woman who is going for her fourth child, well, depending on what method, I don’t even talk to her about it… I think…” (Midwife, > 50 years of age)* *“…It’s the easy way for the woman [to say]: tell me what I have to use and give me it…”, (Family doctor, 41–50 years of age)* *“There are practitioners who decide for the women who attend consultation by presenting them only with what they believe they should choose”, (Midwife, 20–30 years of age)*	

Other practitioners agreeded that the delay in referring users to administer some long-acting contraceptives (IUD or implant), increased the risk of pregnancy, while it hindered the relationship with the practitioner, as women responded badly to the delay.

**Table:** 

***Category: Health system that hinders good monitoring*** *“the fact is that when the woman asks for the visit, she does it on the spur of the moment and wants it right away (IUD or implant) (…) then the fact that it takes a month or takes two… or simply telling her that when she gets her period to call and when she calls all the visits are booked and she has to wait until the next month… that’s a problem… that’s a problem… as also many get angry…”, (Midwife, > 50 years of age)* *“If you have a waiting list of X months for a vasectomy, you have to say that, until surgery, they have to use a method. It’s not the only problem, obviously, but there is a lot of evidence that proves quickstart: if the day they come to the appointment they start the method, treatment adherence is higher than if they have to wait. It’s proven to be the case…”, (Family doctor, > 50 years of age)*	

However, some practitioners expressed that young people are more comfortable talking about the subject in workshops or outside health centres.

They stressed the suitability of DST for contraception, as it would encourage users to attend consultation previously informed of the available methods. They were also of the opinion that the creation of a DST for contraception would allow them to have a space where they could consult information and get up to date.

**Table:** 

***Category: Need to improve accessibility to information for practitioners and users in friendly environments*** *“I’ve often found that they do not want to go straight to the primary care centre, with doctors or nurses… maybe the white coat frightens them (A), I do not know… and they ask you for a more informal practitioner, maybe on more level terms, right? And so often we refer them to health centres, which maybe approach the issue in a more informal way, or there, they also hand out condoms… it’s like a workshop, rather than going straight to the [doctor’s] office… that’s my experience…”, (Social educator, 31–40 years of age)* *“… Always leave the door open for her to call for any questions she might have, right? But above all, what method, what it consists of, what it involves, how it is used, and what to do if something goes wrong.. “, (Midwife, > 50 years of age)* *“…We’re really pushed for time in consultation and having a (DST) tool that, simply and in a short space of time, can help you so that the woman can make the decision… Well, yes, I think it can be useful… (Family doctor, 41–50 years of age).* *“We would use a tool a lot in the day to day. We would share with the patient because s/he’s the one who has to decide… We would explain the pros, the cons and all the options. But the patient is the one who must decide. We give them support, of course… if we have a tool to help us do these things, all the better”, (Family doctor, > 50 years of age)* *“I guess it would be good for this tool to be in different languages: Arabic, English, French, Russian perhaps… and Chinese”, (Midwife, > 50 years of age)*	

## Discussion

The present study has explored the experiences of users and practitioners in relation to contraceptive counselling and identified the perceived needs of both groups. It aimed to constitute a first step towards improving the counselling process.

### Users’ experiences

The vast majority of participants were women who expressed to feel dissatisfied with the advice received due to a lack of information, being dealt with too quickly and/or a feeling of a particular contraceptive method being imposed on them. Their experiences during counselling reflect different styles of healthcare, depending on the practitioner treating them. Those results are similar to a qualitative study conducted in the US which assessed how the choice of contraceptive method was approached in the practitioner’s office [22].

Data analysis principally describes practitioners who advise users without exploring their abilities or difficulties regarding the use of a method, or their knowledge and needs of and/or preferences for contraception. Their request was done without discussion between patent-practitioner. Dehlendorf called that a “*foreclosed approach*”, whereby the suppliers only provide information on the methods users explicitly mention [22]. In Spain, the 20% of users of COC that forgot to take their pill was because they did not know that they had to take it at the same time every day [23]. This fact therefore demonstrates the need for individualized counselling that takes into account the multiple factors that surround these women.

Some of the participants mentioned having received biased information that did not include all contraceptive options. Others described practitioners that imposed the contraceptive method on them, which can be counterproductive, according to a study [24] in which it was found that women who felt under pressure to use a contraceptive implant were more likely to cease to use it. Another study [25] reports that women who had felt under pressure during counselling were less likely to return to a family planning centre in the future.

When information focuses on and is tailored to the needs of the user during counselling, it has positive consequences. Adequate counselling is associated with improved adherence to the contraceptive method chosen and the continued use of a highly or moderately effective method 6 months after the visit [26]. Conversely, when patient preferences are ignored, treatments are seen to be less effective and clinical variability increases unreasonable, which, in this circumstance, is exclusively subject to medical judgement [27].

For over 20 years, a paradigm shift in contraceptive counselling has been promoted towards a model of shared decision-making in which the practitioner. This counselling includes the woman’s medical history, the advantages and disadvantages of all existing contraceptive options, and the preferences of the users. Her concerns, personal and family values are also included in this model [28, 29].

Research into contraception recognizes that women are more satisfied with the advice received and the method chosen when they experience shared decision-making [30]. But in light of all of the above, it seems clear that some practitioners resist and continue to assume a directive role. This may be due to a greater affinity to a paternalistic paradigm, or a feeling of discomfort. This leads them to maintain the informed choice approach, as they do not know how to manage the balance between the priority of patient autonomy in decision-making and the desire to encourage women to use highly efficacious methods [22].

Receiving information on the available methods and the sensation of not feeling judged or forced by the practitioner to choose is positively valued [31]. Women are grateful that practitioners help them make the decision, in a friendly environment, provided that the opinion of the practitioner is accompanied by an underlying explanation [17].

It is essential to establish a good interpersonal relationship during contraceptive counselling, given its personal, sensitive nature [32]. Communication behaviours, like cordially greeting patients and small talk, have been associated with the continuation of the use of the contraceptive method in time [26]. Authors such as Dehlendorf recommend that practitioners should start the conversation by focusing on the user’s preferences. It includes being objective and non-judgemental in an atmosphere of an interactive educational conversation. But without participating in the actual choice of the method, to ensure that the women are not inappropriately influenced [13].

Most participants acknowledged that they lacked information to choose according to their needs. It correlates with the results of the study by Hodgson, in which most participants were not aware of the variety and characteristics of contraceptive options [33].

According to various authors, women wish to receive clear, objective information about the method as well as be corrected with regard to misinformation and/or myths [34]. Some of the women who attended the FG considered “*that COC reduce fertility and that therefore it was necessary to take a break*”, myths that negatively condition the use of COC [35]. In this regard, in 2020 the SEC published that 28% of COC users in our country erroneously take such breaks, increasing the risk of unplanned pregnancy [3]. These evidences appears to be a clear example that some practitioners may not be aware of the myths circulating and/or have insufficient information to counter them.

In a recent publication on counselling [7], it is recommended first to respond to the priorities expressed by the users, in response to the initial question about preferences. For example, if they point out that that the most important aspect of the method the user chooses is not to have to be conscious of it, practitioners should provide the range of options to respond to this demand and start with questions like: “*There are methods that are taken every day, every week, every month, every three months or even less frequently. Which one seems best for your situation?*”*.* Then, it is recommended to review the general characteristics (e.g., effectiveness or the resulting changes to menstruation) and discuss the range of options within each characteristic. Users display strong and varied preferences, as well as erroneous knowledge regarding changes in menstrual bleeding. It is important to stress that the change itself, such as amenorrhea, can be seen as a benefit for some women and as a negative side effect for others [34], hence its importance for exploration.

The user participants called for more information during counselling on the effectiveness of the different methods. It correlates with other studies [9, 10] where effectiveness is considered as a high priority preference for users when choosing a method. However, much of the population is unaware of the absolute and relative effectiveness of the different methods for preventing pregnancy [36].

In line with the findings of other studies [17, 37], users displayed fear and concern regarding possible side effects. Especially when it comes to hormonal methods and their effect on mood, mental well-being and future fertility. Some authors blame the situation on social networks, where negative information on methods is more commonly dealt with than positive information [38–41]. Holt stresses the importance during counselling of informing as to the adverse effects of the method, and reviewing them during follow-up visits [17]. Advanced counselling on possible side effects has been associated with both satisfaction with and adherence to the method [42, 43].

For many young people, sexual debut comes before the acquisition of adequate knowledge about contraception [33]. This situation encourages, as shown in a recent study, that 18% of women, most of them with low socio-economic and educational level, did not use any contraceptive method during their first sexual intercourse [44].

Therefore, it would be necessary to access the social and educational environment of these young people, before their first sexual intercourse, in order to provide them with information and encourage the use of contraceptive methods. A good approach could be the promotion of consultation and use of DST in contraception through cell phones in schools and educational institutions. Young people are a group that is reluctant to go to a contraceptive counselling office, but they are very accepting of mobile applications, which they feel respect their privacy and confidentiality [45]. Several studies report that prior consultation of a DST in contraception facilitated the conversation during the counselling visit, and that several participants expressed a desire to attend the clinician’s office after use [46, 47].

### The opinion of the practitioners

The role of practitioners in contraceptive counselling is key when choosing the method. According to data taken from the 2020 Survey on Contraception in Spain, 64.3% of women of childbearing age have gone to a practitioner to choose the contraceptive method that best suits their needs. Especially adult women, as they give importance to the information received during consultations [3].

Practitioners participating in the study agree that quality counselling should be provided. Some of the elements mentioned considered for a consistent process of shared decision-making were: individual’s preferences and values, stressing the importance of creating a close, trusting relationship in an environment of privacy, respect, empathy and non-discrimination on the grounds of gender, race/ethnicity, social class or other factors. All those elements are in line with the reports of many author [8, 22, 48]. However, they indicated that, in practice, it is difficult for them to explore knowledge or needs and inform of all available methods, arguing, among other things, communication barriers with the user due to language and/or cultural aspects. These difficulties must raise the alarm since they can lead to what Downing has called “*intersections of ethnicity and social class in provider advice regarding reproductive health*” and are reflected in studies showing bias in counselling according to the race and/or culture of the user. In this regard, women of colour are more likely to receive advice to limit their childbearing than white women [49], while at the same time they are more susceptible to being pressured by the practitioner to use highly effective contraceptive methods [50].

We must also consider the fact that the participating practitioners felt that not all of them offer advice with updated evidence-based information. It collapses with the lack of specific training in contraception in some cases, and a lack of communication skills. It encourages a paternalistic approach to healthcare directed towards the choice of the method they know best.

In contrast with directive counselling, shared decision-making provides a structure for counselling that protects against perceived or actual prejudice in counselling. It is focused explicitly on the preferences expressed by women. However, since such bias may influence the way in which support is provided to decision-making, practitioners should be aware of the possibility that it may subtly influence their counselling and should work on not overemphasizing specific methods based on the assumption of what “they should want” [7].

DSTs facilitate information and counselling by allowing the practitioner and the user to freely access them from any digital device. This process facilitates personal reflection before making the decision that best suits their preferences, needs and state of health [15, 51, 52].

Given the findings of this study, the need is identified for health institutions to weigh up the short- and long-term advantages of contraceptive counselling backed up with a DST for contraception. It may seem that the use of a DST consumes consultation time that is not available. However, a first visit with the support of a DST could solve some of the shortcomings in counselling detected. As well as practitioners’ lack of knowledge, their poor approach to crucial issues, unexplored false beliefs and/or cultural or language barriers, that could be improved with a translation and cultural adaptation of the DST for users of diverse backgrounds.

Based on a pilot study, we are currently evaluating whether the use in consultation of “SHARECONTRACEPT”, a DST in hormonal contraception, corresponding to the 1st Phase of the “Contraception” DST, improves adherence to treatment, user satisfaction, decisional conflicts, counsellor or clinician satisfaction, and the knowledge acquired. This research project has received a grant from the European Regional Development Fund (ERDF) through competitive call FIS 18 for Health research projects, of the Carlos III Health Institute and has been recently published [53].

However, the promotion of DST must be accompanied by actions and/or courses to help practitioners improve their knowledge of contraception as well as their communication skills. These measures have achieved very good results regarding problems of the health system such as excessive caseloads, the lack of experience on the part of clinicians, and a lack of material resources and/or personnel [54, 55].

## Limitations

The present study does present some limitations. The sampling of this study was for convenience, so it may not be representative. Our study was open to all individuals with experiences in contraceptive counselling, so it could be that the users and professionals who agreed to participate in the research had unmet needs, complaints, or doubts during contraceptive counselling.

Moreover, the study excluded individuals who do not speak and understand Spanish, who represent a sizeable portion of the immigrant people in Tarragona, and likely face even more significant barriers to managing their contraceptive decisions.

## Conclusions

Users are dissatisfied with the counselling they have received and demand truthful and objective information about the effectiveness and/or side effects of all contraceptive methods, among others, in order to improve their knowledge. They value an approachable attitude of the professionals who show interest in their needs, values and priorities.

Some practitioners are seen as being reluctant to assume a process of shared decision-making during counselling due to their lack of up-to-date knowledge of contraception, lack of communication skills and/or their perceived overload.

Free access to a DST on contraception, from a computer or cell phone, can provide the population with information accessible from home, updated and based on the best evidence, at the click of a button, which is very well accepted by young people. It seems to have been demonstrated that its use, before going to the clinic, can facilitate conversation with the clinician, as well as motivate them to want to go to a contraceptive counselling clinic after its use.

Healthcare institutions should consider providing more time for consultation for DST contraception counselling, which could yield savings in the long run as a result of increased treatment adherence and satisfaction, and fewer voluntary termination of pregnancies (VTPs), between others.

## Data Availability

The datasets produced and/or analysed during the current study are not openly available. They are located on a data storage platform (OneDrive) owned by the Universitat Rovira i Virgili that can only be accessed by researchers with their credentials, but are available from the corresponding author on reasonable request.

## Abbreviations

COC: Combined oral contraception
STI: Sexually transmitted infections
SEC: Spanish Society of Contraception
LARC: Long-Acting Reversible Contraception
IUD: Intrauterine devices
QCC: Quality on contraceptive counseling
DST: Decision support tool
FG: Focus groups
US: United States
VTP: Voluntary termination of pregnancy
